# Case Report: Multidisciplinary collaboration for the treatment of severe necrotizing fasciitis in the perineum caused by rectal cancer perforation

**DOI:** 10.3389/fsurg.2026.1790699

**Published:** 2026-05-15

**Authors:** Hang Hu, Wen Zou, Xiaoqian Luo, Shaojun Yu, Jianwei Wang, Xinlei Hu, Feng Yu, Ran Ji

**Affiliations:** 1 Department of Burns and Wound Center, The Second Affiliated Hospital, Zhejiang University School of Medicine, Hangzhou, Zhejiang, China; 2 Department of Surgery ICU, The Second Affiliated Hospital, Zhejiang University School of Medicine, Hangzhou, Zhejiang, China; 3 Department of Colorectal Surgery and Oncology (Key Laboratory of Cancer Prevention and Intervention, China National Ministry of Education), The Second Affiliated Hospital, Zhejiang University School of Medicine, Hangzhou, Zhejiang, China; 4 Center for Medical Research and Innovation in Digestive System Tumors, Ministry of Education, Hangzhou, China; 5 Department of Orthopedic Department, The Second Affiliated Hospital, Zhejiang University School of Medicine, Hangzhou, Zhejiang, China

**Keywords:** Fournier's gangrene, multidisciplinary care, patient-centered care, rectal cancer perforation, staged surgical management

## Abstract

Fournier's gangrene (FG) is a rare but life-threatening necrotizing fasciitis of the perineum and genital regions. Rectal cancer perforation is an uncommon cause of FG, and optimal management remains challenging.

**Case presentation:**

We report a 62-year-old male with rectal cancer complicated by severe FG secondary to rectal perforation. The patient underwent emergency fecal diversion, repeated extensive perineal debridement combined with vacuum-assisted closure (VAC) therapy, and intensive supportive care. Following infection control and clinical stabilization, definitive surgical management was performed, including laparoscopic abdominoperineal resection and reconstruction of the large perineal defect using a vascularized gracilis muscle flap and a gluteus maximus fascia composite flap. At two-month follow-up, the patient demonstrated complete wound healing, stable colostomy function, and recovery of independent daily activities.

**Conclusions:**

This case highlights the complexity of managing FG associated with rectal cancer perforation and demonstrates the feasibility of sequential treatment strategies integrating infection control, oncologic resection, and reconstructive surgery.

## Background

FG is a rapidly progressing bacterial infection primarily involving the subcutaneous tissue and deep fascia while sparing the underlying muscle (1). It most commonly affects the scrotal, perianal, and perineal regions and is characterized by rapid spread, systemic toxicity, and a high risk of mortality if not promptly treated (2).

FG secondary to rectal cancer is rare, and its precise incidence remains unclear due to the limited number of reported cases (3). Rectal cancer perforation is a recognized risk factor for the development of FG in such cases. To date, only few cases of FG associated with rectal cancer perforation have been reported, and optimal management strategies remain controversial (4).

Standard management of FG consists of early diagnosis, aggressive surgical debridement of necrotic tissue, and broad-spectrum intravenous antibiotic therapy (5). When FG is complicated by rectal cancer, treatment becomes substantially more complex, as infection control must be balanced against the need for definitive oncologic resection. In this setting, determining the appropriate timing and extent of tumor-directed surgery represents a major clinical challenge. Moreover, extensive soft-tissue destruction frequently necessitates staged reconstructive procedures to restore perineal integrity and function after infection control.

Here, we report a case of rectal cancer perforation complicated by severe FG that was successfully managed through a patient-centered, multidisciplinary, and staged therapeutic strategy. This case highlights the challenges inherent in coordinating infection control, physiological stabilization, oncologic resection, and reconstruction in a rare but highly complex clinical scenario. This case is presented not only to describe a rare clinical entity, but also to illustrate how surgical decision-making can be aligned with the dynamic physiological trajectory of a critically ill patient.

## Case presentation

A 62-year-old male was diagnosed with rectal cancer by undergoing a colonoscopy because of intermittent rectal bleeding for about 5 months at the local hospital in September 2023. His medical history was unremarkable except for surgical treatment for an inguinal hernia three years prior.

The patient was subsequently referred to another cancer hospital, where PET-CT revealed a solitary metastasis in the left lung without evidence of other distant disease. Radiation therapy targeting the lung metastasis (60Gy/8f) was initiated on December 28, 2023, followed by radiation therapy for rectal cancer (45Gy/25f) and three cycles of XELOX chemotherapy. The patient later transferred to our hospital, where he received an additional three courses of XELOX chemotherapy combined with bevacizumab. The last cycle of bevacizumab was administered on April 30, 2024.

On May 15, 2024, the patient sought care at a local hospital due to reduced anal defecation, difficulty in passing stool, and worsening buttock pain. These symptoms progressed, and he was admitted to a cancer hospital for symptomatic management. On May 19, 2024, he was transferred to the emergency department of our hospital.

**Figure 1:**
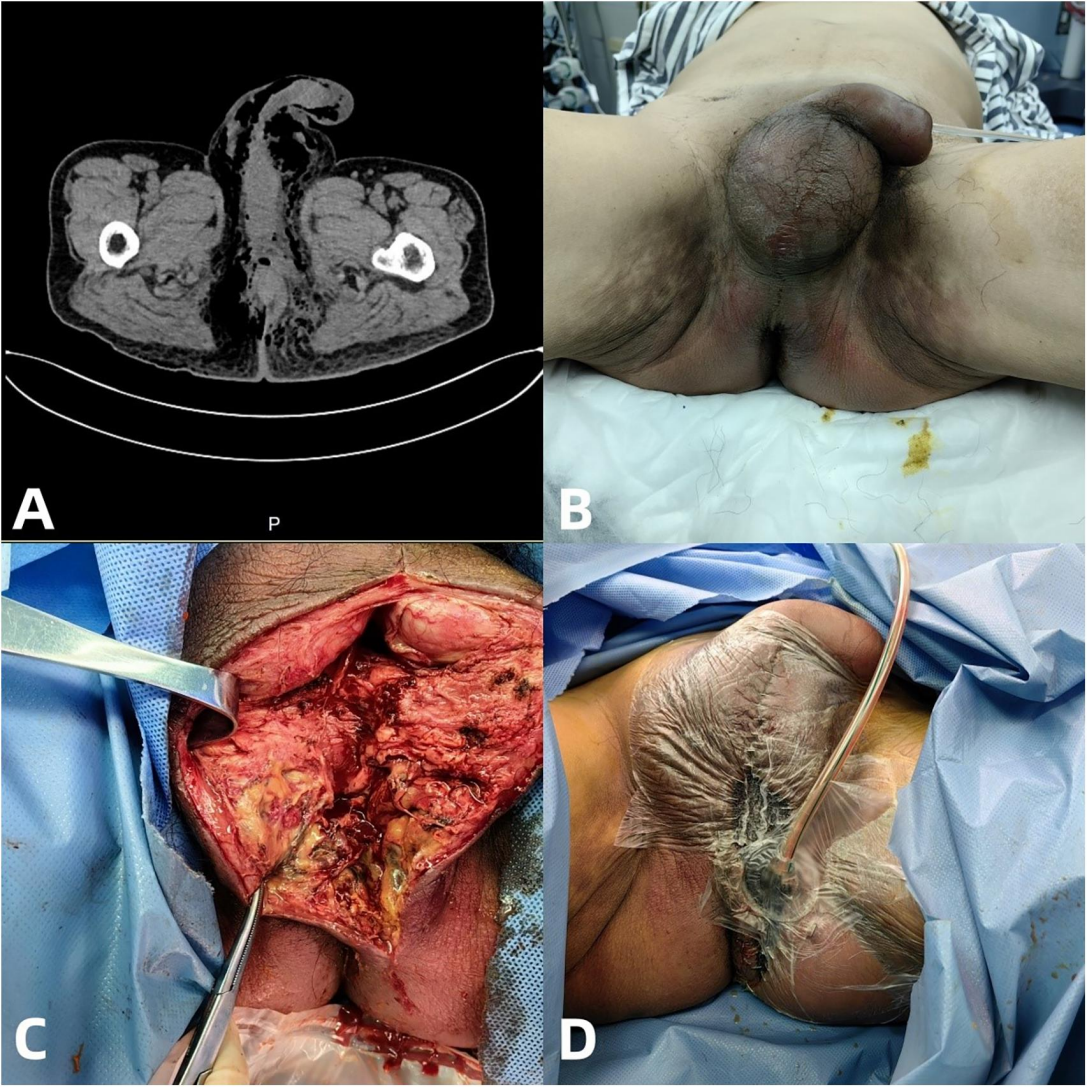
**(A)** Emergency enhanced CT scan of the entire abdomen indicates a discontinuity in the left wall of the rectum, with surrounding exudate, fluid accumulation, thickening of the fascia around the rectum, exudate, and gas accumulation around the pelvic floor and perineum, and multiple gas accumulations in the groin area, pelvic wall, and left abdominal cavity on both sides. **(B)** Physical examination revealed extensive redness and swelling around the scrotum and anus, extending all the way to both buttocks and at the base of the thighs. The scrotum and penis were also highly swollen. **(C)** During the emergency perineal incision and drainage surgery, the scrotum and perineal anal soft tissue were fully drained, and a large amount of feces and pus were extracted. **(D)** Vacuum assisted closure (VAC) after perineal incision and drainage surgery.

An enhanced CT scan revealed a discontinuity in the left rectal wall, with surrounding exudate, fluid collection, thickened fascia, gas accumulation in the pelvic floor, and extensive gas presence in the groin and pelvic cavity (Figure 1A). Physical examination revealed severe redness, swelling, and necrosis around the scrotum, anus, and thighs, with pronounced scrotal and penile edema (Figure 1B). Laboratory findings showed a white blood cell count of 6.3 × 10⁹/L and elevated C-reactive protein (CRP) at 278.6 mg/L.

On May 20, 2024, due to severe infection and critical condition, the patient underwent emergency “temporary transverse loop colostomy with perineal incision, extensive debridement, drainage, and vacuum-assisted closure (VAC) therapy.” Extensive drainage of necrotic tissue from the scrotum and perineal region revealed large amounts of feces and pus, followed by vacuum-assisted closure (VAC) application (Figures 1C,D). Postoperatively, the patient was admitted to the surgical ICU for intensive care and anti-infective therapy. Empirical broad-spectrum antimicrobial therapy with imipenem–cilastatin combined with teicoplanin was initiated on admission. Microbiological cultures from wound secretions and drainage fluid revealed polymicrobial infection, including Escherichia coli and Enterococcus species initially, followed by Klebsiella pneumoniae and later methicillin-resistant Staphylococcus epidermidis during the course of treatment. Antibiotic therapy was adjusted according to susceptibility testing and was subsequently de-escalated to piperacillin–tazobactam on May 29 following clinical stabilization. Inflammatory markers, including C-reactive protein and procalcitonin, showed a progressive decline, accompanied by stabilization of body temperature and hemodynamic status, consistent with resolution of sepsis. These improvements occurred in parallel with repeated surgical debridement and vacuum-assisted closure (VAC) therapy.

Subsequent perineal debridement and VAC procedures were performed on May 22, 25, and 27. On May 29, the incision was temporarily sutured, and the patient was transferred to ICU to prepare for radical rectal cancer resection.

**Figure 2:**
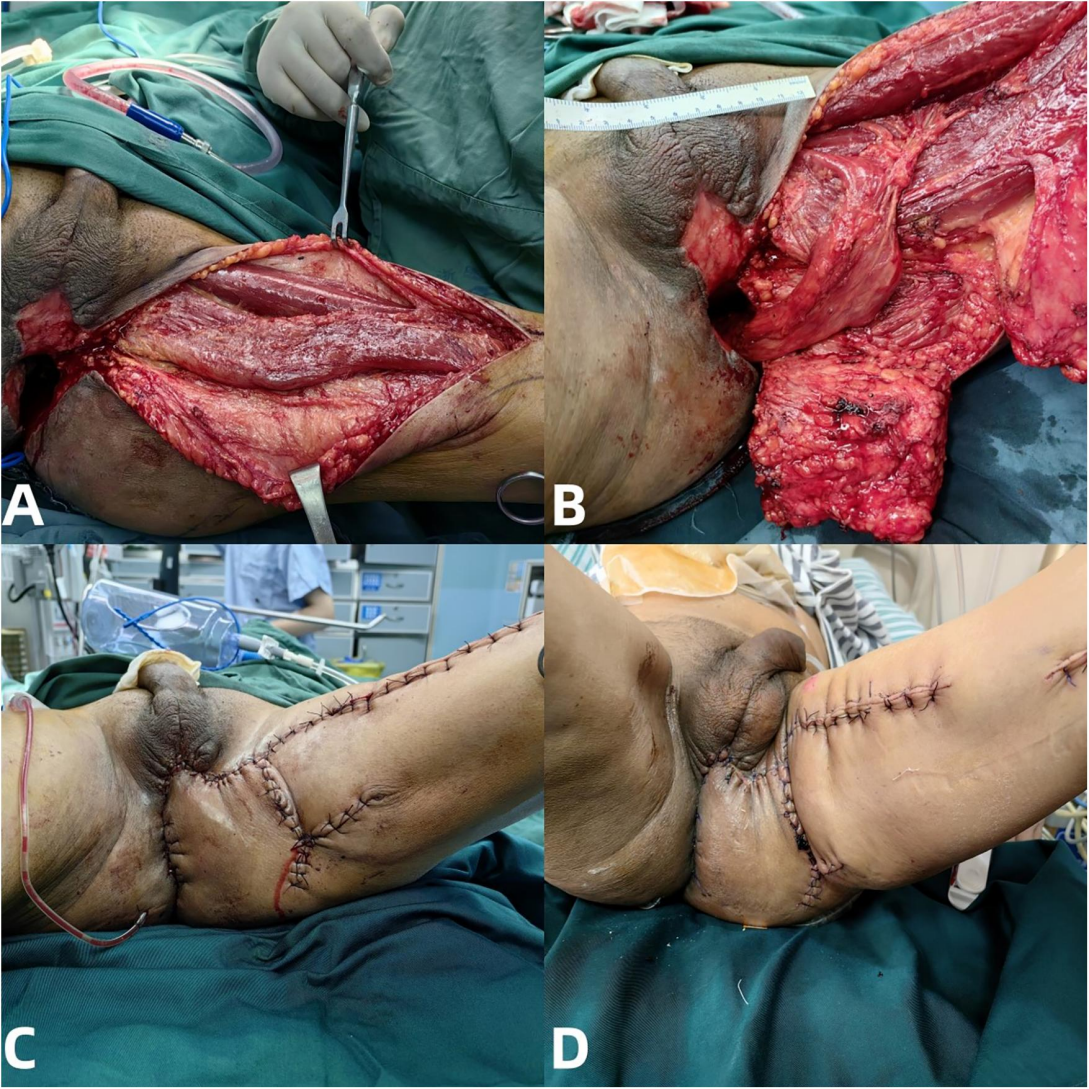
**(A)** June 1st, due to the patient taking a large amount of food after removing the endotracheal tube, a large amount of feces gushed out from the perineum again. **(B)** Permanent colostomy of the transverse colon in the abdomen was performed by a colon surgeon. **(C)** The postoperative pathological report of Miles surgery showed moderately differentiated adenocarcinoma (rectal issue), pTNM (AJCC 8th edition): ypT3N0Mx. **(D)** The defect at the excision site of the perineum and rectum, a perineal sinus tract was left after abdominal perineal rectal resection.

On June 1, after extubation and dietary intake, fecal discharge from the perineal incision necessitated an emergency debridement and negative-pressure wound therapy. A permanent transverse colostomy was performed to ensure complete fecal diversion and reduce ongoing contamination of the perineal wound in preparation for definitive surgery (Figures 2A,B). On June 13, the patient was transferred to the colorectal surgery department, and after clinical stabilization, with stable vital signs and significant reduction in inflammatory markers, and re-evaluation, the tumor was considered unsuitable for sphincter preservation, and abdominoperineal resection (APR) was indicated. On June 19, he underwent laparoscopic abdominoperineal resection (APR, Miles surgery). Postoperative pathology revealed moderately differentiated adenocarcinoma (ypT3N0Mx), with negative proximal, distal, and circumferential margins. A total of 2 lymph nodes were examined, all of which were negative (0/2). Histopathological evaluation demonstrated partial tumor regression (TRG2) (Figure 2C).

**Figure 3:**
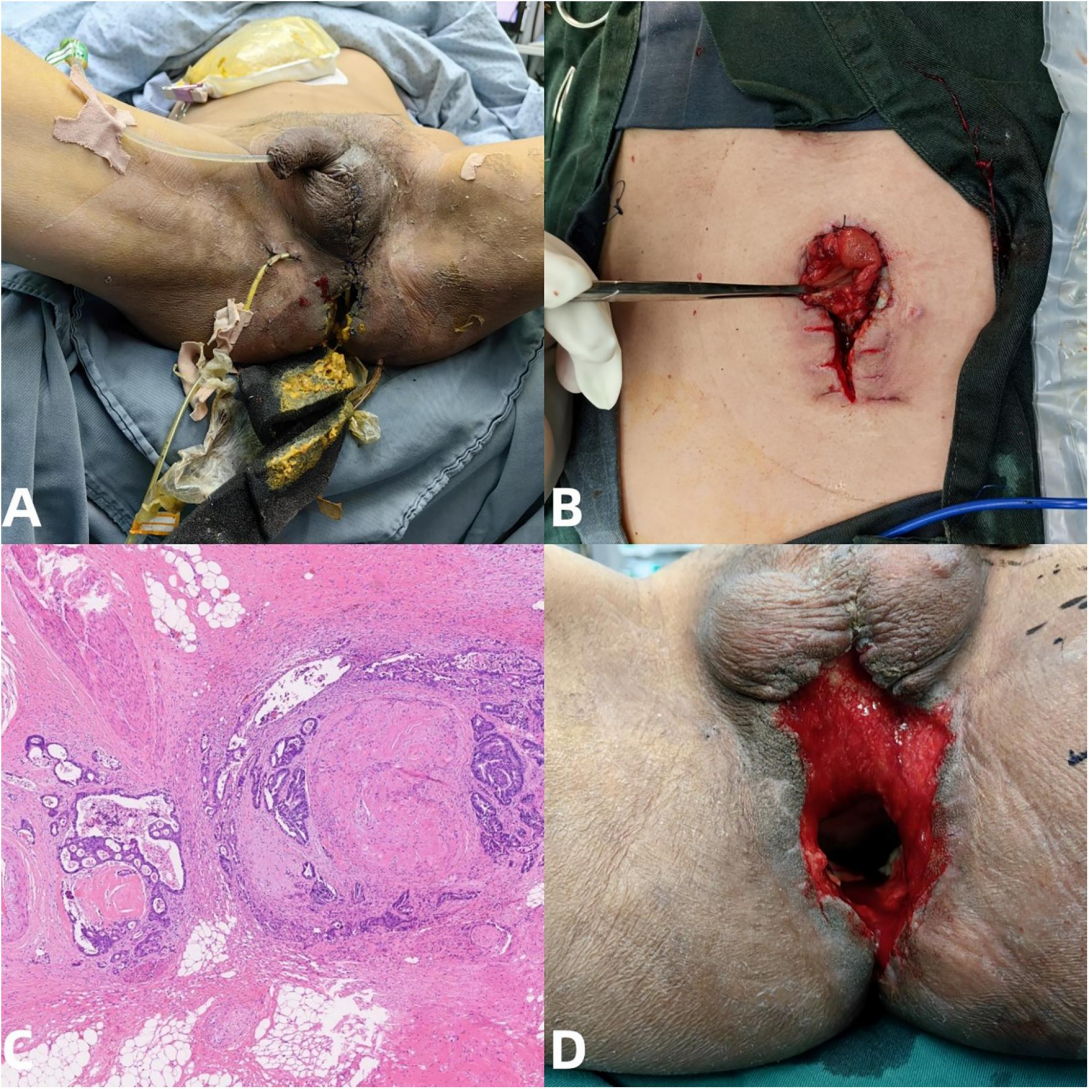
**(A)** Longitudinal incision on the inner side of the patient's left thigh, showing the gracilis muscle and its nutrient vessel—the gracilis muscle branch of the deep femoral artery. **(B)** Filling of the perineal sinus tract with a vascularized gracilis muscle flap and prepare to transplant a composite tissue flap of gluteus maximus fascia to cover the perineal wound. **(C)** All skin flap transplants have been completed, the perineal and thigh incisions have been sutured, and the surgery has been successfully concluded. **(D)** Two days after surgery, the patient recovered well and all incisions were dry and healthy. The patient was allowed to be discharged and returned to the local hospital for routine dressing change treatment.

Following APR, the patient required further treatment for a significant perineal defect. On July 2, he was transferred to the Burn and Wound Repair Department. Between July 3 and 16, multiple sessions of perineal debridement and VAC therapy were performed until the wound bed was clean. On July 19, under general anesthesia, the defect was repaired using a vascularized gracilis muscle flap and a gluteus maximus fascia composite flap (Figures 3A–C). The patient recovered well postoperatively, with all incisions healing satisfactorily. He was discharged on July 22 to continue dressing care at a local hospital (Figure 3D).

**Figure 4:**
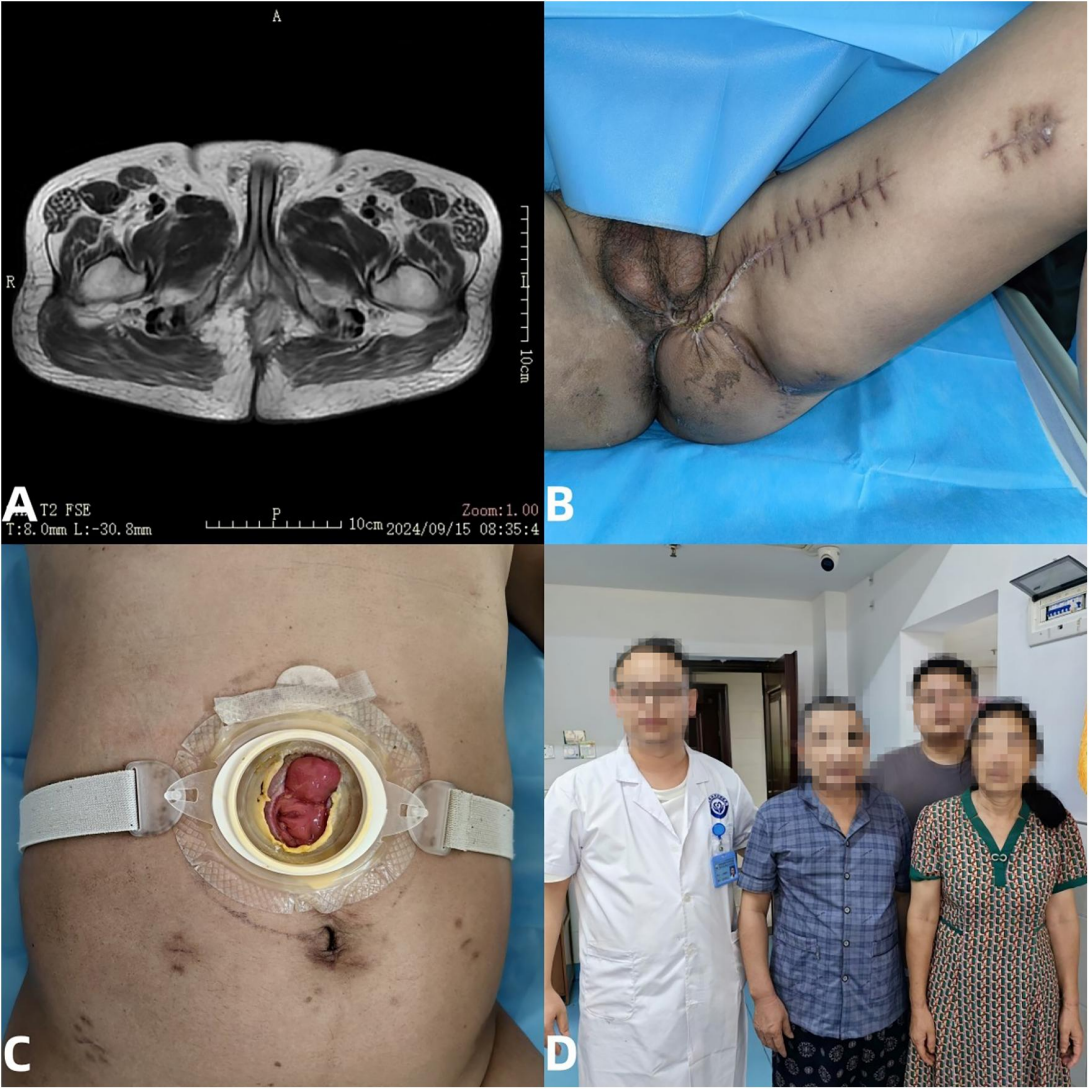
**(A)** 2-month follow-up after surgery, the pelvic MRI of the patient shows that the perineal rectal resection cavity is completely filled with the gracilis muscle, and there is no residual or recurrent rectal cancer. **(B)** The patient's perineal area wound completely healed. **(C)** The abdominal transverse colostomy defecation was good. **(D)** His daily life was completely self-sufficient, and their weight increased by 5KG compared to the emergency department in May 2024.

During the early two-month follow-up on September 15, 2024, pelvic MRI showed complete filling of the perineal rectal resection cavity with the gracilis muscle flap and no evidence of residual or recurrent cancer. The perineal wound had healed completely, the abdominal colostomy was functioning well, and the patient had gained 5 kg since May 2024. He resumed normal daily activities with full self-sufficiency (Figures 4A–D). At the latest follow-up (22 months), the patient remains alive, with sustained wound healing and satisfactory colostomy function. However, further oncologic assessment was limited, as the patient declined additional evaluation and treatment of the lung metastasis.

## Discussion

Fournier's gangrene secondary to rectal cancer perforation represents a rare and highly challenging clinical scenario, characterized by the coexistence of severe infection, malignancy, and physiological instability. In such cases, management requires balancing effective source control with the need for definitive oncologic treatment, often under conditions of limited physiological reserve. In the present case, successful management relied on prioritizing infection control through repeated debridement and fecal diversion, followed by gradual progression to definitive resection and reconstruction as the patient's condition improved. This sequence reflects commonly accepted principles in the care of critically ill surgical patients, where the extent and timing of intervention are adjusted according to the patient's clinical status.

Rather than representing a novel strategy, the management approach in this case illustrates the practical application of well-established surgical principles. In particular, staged intervention allowed initial control of sepsis and local infection, creating the conditions necessary for subsequent oncologic resection and reconstruction. Importantly, the transition from infection control to definitive surgery was guided by overall clinical improvement, including stabilization of systemic condition and recovery from the acute septic phase. This stepwise progression is consistent with routine clinical decision-making in complex surgical infections, although it is not always explicitly described in the context of malignancy-associated Fournier's gangrene not always explicitly described in the context of malignancy-associated Fournier's gangrene. Effective infection control, including timely antimicrobial therapy and repeated debridement, was essential in stabilizing the patient and enabling subsequent definitive surgical management. Compared with previously reported cases (6–8), our approach emphasizes early fecal diversion for infection control, followed by staged progression to definitive oncologic resection and reconstruction. Although derived from a single case, this approach may be applicable to other complex surgical infections complicated by malignancy and severe physiological derangement.

From the outset, definitive oncologic resection was recognized as the theoretical endpoint of treatment given the underlying rectal cancer. However, this objective was not assumed to be immediately attainable. Rather than adhering to a fixed surgical timeline, the treatment strategy followed a physiology-driven and time-dependent pathway. In the early phase, when profound sepsis and physiological instability predominated, only procedures with minimal physiological burden—such as fecal diversion and aggressive debridement—were considered acceptable (9). As infection control was achieved and organ function gradually recovered under ICU-supported management, the range of feasible surgical options expanded accordingly. Radical abdominoperineal resection was therefore neither delayed arbitrarily nor pursued according to a predefined schedule, but was performed once repeated reassessment confirmed that the patient's physiological reserve had reached the threshold required to tolerate definitive oncologic surgery.

Determining the optimal timing for definitive oncologic intervention in this context required an integrated, ICU-guided assessment rather than a checklist-based evaluation of isolated parameters. In the present case, sustained hemodynamic stability and effective control of systemic infection functioned as non-negotiable gatekeeping conditions; without stability in these domains, escalation to radical surgery was considered unsafe regardless of local wound status. Once these prerequisites were satisfied, attention shifted to factors that modulate surgical tolerance, including nutritional reserve, functional recovery, and the trajectory of systemic inflammation (10). Importantly, these variables were not weighted equally but interpreted in relation to the anticipated physiological burden of the planned procedure. As the patient's physiological reserve progressively improved under ICU management, the spectrum of feasible surgical options expanded in parallel. Radical abdominoperineal resection was undertaken only when repeated reassessment confirmed not merely the absence of prohibitive instability, but sufficient global reserve to tolerate both oncologic resection and subsequent complex reconstruction. This stepwise, weighted decision-making process illustrates how ICU-supported evaluation enables alignment between surgical timing and evolving physiological capacity, rather than reliance on arbitrary temporal thresholds.

Although several scoring systems, such as the Fournier's Gangrene Severity Index, have been proposed to assess disease severity, their role in guiding the timing of definitive oncologic intervention remains limited. In practice, clinical judgment based on the overall trajectory of the patient's condition remains central to decision-making.

## Conclusion

Fournier's gangrene secondary to rectal cancer perforation is a rare and life-threatening condition that requires complex and coordinated management. This case demonstrates that successful outcomes can be achieved through timely infection control, appropriate use of fecal diversion, and subsequent definitive oncologic resection and reconstruction. Rather than representing a novel treatment paradigm, this experience reflects the application of established surgical principles in a particularly challenging clinical context. Multidisciplinary collaboration remains essential in managing such cases.

## Data Availability

The raw data supporting the conclusions of this article will be made available by the authors, without undue reservation.
